# Complying with the Guideline for Quality and Equivalence for Topical Semisolid Products: The Case of Clotrimazole Cream

**DOI:** 10.3390/pharmaceutics13040555

**Published:** 2021-04-14

**Authors:** Teresa Alves, Daniel Arranca, Ana Martins, Helena Ribeiro, Sara Raposo, Joana Marto

**Affiliations:** 1 Research Institute for Medicine (iMed.ULisboa), Faculty of Pharmacy, Universidade de Lisboa, 1649-003 Lisbon, Portugal; teresaalves@campus.ul.pt (T.A.); amartins@farm-id.pt (A.M.); hribeiro@campus.ul.pt (H.R.); sraposo@edol.pt (S.R.); 2 Laboratório Edol—Produtos Farmacêuticos, S.A., 2795-225 Linda-a-Velha, Portugal; darranca@edol.pt

**Keywords:** generic medicine, pharmaceutical development, quality by design, rheology, topical delivery system

## Abstract

Semisolids constitute a significant proportion of topical pharmaceutical dosage forms available on the market, with creams being considered profitable systems for releasing active substances into the skin. This work aimed at the development of a generic Clotrimazole topical cream, based on the assumptions that assist the development of such formulations. First, the critical parameters to obtain a final formulation as similar as possible to the reference product were defined. Then, the percentages of cetyl palmitate and octyldodecanol were identified as critical variables and chosen for optimization in further studies. A “quality by design” approach was then used to identify the effect of process variability on the structural and functional similarity (Q3) of the generic product qualitatively (Q1) and quantitatively (Q2). A two-factor central composite orthogonal design was applied and eleven different formulations were developed and subjected to physicochemical characterization and product performance studies. The results were used to estimate the influence of the two variables in the variation of the responses, and to determine the optimum point of the tested factors, using a design space approach. Finally, an optimized formulation was obtained and analysed in parallel with the reference. The obtained results agreed with the prediction of the chemometric analysis, validating the reliability of the developed multivariate models. The in vitro release and permeation results were similar for the reference and the generic formulations, supporting the importance of interplaying microstructure properties with product performance and stability. Lastly, based on quality targets and response constraints, optimal working conditions were successfully achieved.

## 1. Introduction

Semisolids constitute a significant proportion of pharmaceutical dosage forms. Semisolids for cutaneous application may be distinguished according to the following categories: ointments, creams, gels, pastes, medicated plasters, or cutaneous patches. According to their structure, ointments, creams, and gels generally show viscoelastic behaviour and are non-Newtonian [1]. Among the different topical dosage forms available in the market, cream formulations continue to receive growing attention as profitable systems to deliver drugs and cosmetic agents into the skin [2].

In 2018, the European Medicines Agency (EMA) released a draft guideline on the quality and equivalence of topical products, stating that the bioequivalence and pharmaceutical equivalence of the generic drug must be ensured during the drug development process, before reaching the stage of therapeutic equivalence studied in clinical trials. Thus, performance tests must be carried out on the generic drug [3,4,5]. Pharmaceutical equivalence of a reference-listed drug (RLD) and a generic product means that their active pharmaceutical ingredient(s) (API)(s), dosage form, route of administration, and strength are the same. Currently, for most topical products, demonstration of pharmaceutical equivalence is usually not sufficient to predict therapeutic equivalence. Thus, an extended concept of pharmaceutical equivalence was developed, based on comparative quality data with the relevant RLD, including qualitative and quantitative composition, microstructure, physical properties, product performance, and administration. An additional measure of equivalence is the product performance, evaluated by in vitro experiments using artificial membranes and/or human skin to determine the rate and extent of drug release and permeation [6]. As for all medicines, the safety of generic medicines continues to be monitored after authorization [7].

In the pharmaceutical industry, there is a continuous demand for the development of new products and processes, and for the improvement of existing ones, to meet the increasing quality and standards requirements [8]. Pharmaceutical development aims to design a quality product and its manufacturing process to consistently deliver the intended performance [9]. In order to achieve these challenging goals, a quality by design (QbD) approach can be used to support and accelerate the pharmaceutical development and optimize novel formulations. In QbD, it is essential to understand the impact of raw material attributes and process parameters on the critical quality attributes (CQAs) as well as to identify and control sources of variability, in order to mitigate failures. However, pharmaceutical products and processes are complex and multivariate. Understanding the relevant multi-factorial relationships among formulation parameters, process variables and product quality attributes, usually requires the use of multivariate approaches, such as statistical design of experiments (DoE) and multivariate data analysis (MVDA) [10]. In an effort to provide guidance to the pharmaceutical companies, the International Conference on Harmonization of Technical Requirements for Registration of Pharmaceuticals for Human Use (ICH) created the guidelines Q8, Q9, and Q10. The Q8 (R2) Pharmaceutical Development describes the scope and principles of QbD and suggests examples and methodologies for the formulation and process development, ICH Q9 Quality Risk Management provides guidance to implement quality risk management into product development, and ICH Q10 Pharmaceutical Quality System provides guidance for using Q8 and Q9 principles in regulatory strategies [11].

The study reported herein aimed to develop a generic Clotrimazole topical cream qualitatively and quantitatively similar to a reference medicine. To reach the best formulation, variable factors considered critical were studied using a QbD approach. The development of a suitable cream product with physicochemical characteristics and in vitro efficacy similar to the reference formulation (RF) was the key success indicator.

## 2. Materials and Methods

### 2.1. Materials

The Clotrimazole, benzyl alcohol and octyldodecanol were kindly provided by Laboratório Edol—Produtos Farmacêuticos, S.A. (Linda-a-Velha, Portugal). The sorbitan stearate and polysorbate 60 were purchased from Seppic Italia Srl (Milan, Italy). The cetyl palmitate and cetostearyl alcohol were purchased from Mosselman (Ghlin, Belgium). All the solvents were HPLC grade. Purified water was obtained by reverse osmosis and electrodeionization (Millipore, Burlington, MA, USA).

### 2.2. Methods

**Figure 1:**
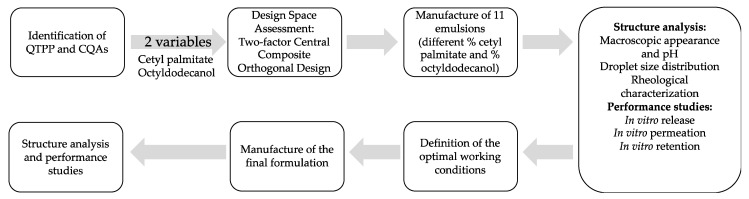
Flowchart including all experimental steps. QTPP—Quality Target Product Profile; CQAs—Critical Quality Attributes.

The methodology followed in this work is summarized in Figure 1. In the next subsections, all of these steps are detailed.

#### 2.2.1. QbD Approach-Identification of Quality Target Product Profile (QTPP) and Critical Quality Attributes (CQAs)

The QTPP describes the desired product quality profile. This parameter establishes the quality characteristics of the cream that should ideally be achieved considering the target, efficacy, and safety of the medicine. The intended therapeutic objective and the purpose of the medicine must also be stated, and it must be explained how these objectives are achieved through product design [5].

Potential CQAs are a set of QTPP that should be within an appropriate range to ensure cream quality achievement. The first step to identify CQAs is to systematically gather all the possible factors that could influence product quality. For this purpose, an Ishikawa diagram was constructed [12]. The viscosity, oscillatory measurements, drug release, and permeation were defined and further delineated to identify potential risks. Two variables were identified for optimization in the subsequent studies.

#### 2.2.2. QbD Approach—Design Space (DS) Assessment

To optimize the emulsion formulation, a two-factor Central Composite Orthogonal (CCO) design was performed using MODDE^®®^ Software (Umetrics, Sweden) [13].

According to preliminary studies (results not shown) and risk analysis (described in 2.2.1), the cetyl palmitate and octyldodecanol were identified as the raw materials that most significantly affect cream CQAs, so the independent variables for formula optimization were the percentages of these two excipients. This design required 11 experimental runs, including three replicated center points for a more uniform estimation of the prediction variance over the entire DS (Supplementary Table S1). Data were analysed using the MODDE^®®^ Software and effects were considered significant when the estimated *p* values were lower than 0.05 (the chosen alfa error), to increase statistical power. The following mathematical quadratic model (Equation (1)) was fitted to the data:

(1) $$Y=\beta_{0}+\beta_{1}{\;X}_{1}+\beta_{2}X_{2}+\beta_{12}X_{1}X_{2}+\beta_{11}X_{1}^{2}+\beta_{22\;}X_{2}^{2}$$

This model describes the zero and second-order effects as well as the interactions between the independent variables.

#### 2.2.3. Manufacturing Process

The oily and the aqueous phases were prepared separately and heated to (75 ± 2) °C with continuous agitation. Clotrimazole, at 2% (*w*/*w*), was dispersed in the melted oily phase. When the two phases reached approximately the same temperature, the oily phase was slowly added to the aqueous phase and stirred at 12,000 rpm for five minutes with the T25 Digital Ultra-Turrax (IKA, Staufen, Germany). Further, the formulations were stirred using a vertical stirrer (IKA^®^ EUROSTAR 60, digital, Staufen, Germany) at 400 rpm until reaching a temperature of 35 °C. Then, the preservative was added and stirred until the mixture was homogeneous. The final pH was adjusted to between 5.5 and 6.5 with HCl (10%, *w*/*v*) or NaOH (40%, *w*/*v*).

#### 2.2.4. Structure Analysis of Emulsions

##### Macroscopic Appearance and pH Determination

The macroscopic appearance of each formulation was visually analysed and used as a first stability indicator. All formulations developed during pre-formulation studies were subjected to an accelerated stability test, with three centrifugation cycles at 10,000 rpm, 5 min each (Heraeus Sepatech Centrifuge, Biofuge), at room temperature, one day after production. The pH values of each formulation were determined by potentiometry using a pH meter (Metrohm pH Meter 744, Metrohm AG, Herisau, Switzerland) equipped with a glass electrode at (20 ± 5) °C.

##### Droplet Size Distribution

Droplet size distribution analysis was carried out at room temperature by light scattering using a Malvern Mastersizer 2000 (Malvern Instruments, Malvern, UK) coupled with a Hydro S accessory. The sample was dispersed in distilled water. The diluted sample was loaded dropwise into the sample chamber containing about 150 mL of water (using a stirrer at 1250 rpm) until laser obscuration between 10% and 20%. Distributions were determined in triplicate for each sample.

The size data were expressed in terms of the relative distribution of the volume of droplets and given as diameter values corresponding to percentiles of 10%, 50%, 90% and span value (mean ± SD). The span value is a statistical parameter useful for characterizing the wideness of the droplet size distribution according to the following Equation (2):

(2) $$Span=\frac{d\left(90\right)-d\left(10\right)}{d\left(50\right)}$$

##### Rheological Characterization

The rheological analysis of all emulsions was performed using a Kinexus Lab+ Rheometer (NETZSCH-Gerätebau GmbH, Selb, Germany) equipped with a universal temperature controller and a cone/plate geometry with a cone diameter of 40 mm and a cone angle of 4°. About 1 g of each formulation was placed on the lower plate before slowly lowering the upper geometry. All measurements were made at 25 °C and samples were analysed one week after manufacturing. Each test was performed at least in triplicate (*n* = 3). Data were evaluated with the rSpace software (NETZSCH-Gerätebau GmbH, Selb, Germany). All the performed tests are described below.

##### Rotational Measurements

Rotational viscosity measurements were performed with a shear rate between 0.1 and 10 s^−1^ with 10 samples per decade. Flow curves were generated by ramping the shear rate from 0.1 to 10 s^−1^ for 5 min with 10 samples per decade. The yield stress was carried out at shear stress from 0.01 to 10 s^−1^ during 5 min with 10 samples per decade. The thixotropic profile was quantified by area differences of the hysteresis loop between the ramp up and ramp down of a flow curve. The areas under the ramp up data points and under the ramp down data points, as well as the hysteresis area, were analysed.

Representative mathematical models (Power Law, Bingham, Herschel-Bulkley, Casson, Cross and Sisko; Supplementary Table S2) were fitted to viscosity measurements, and the best fitting was based on the correlation coefficient (observed vs. predicted) and chi-square value.

##### Oscillatory Measurements

Initially, the linear viscoelastic region (LVR) was estimated from the amplitude sweep test, conducted in stress ranging from 0.1 to 500 Pa, at a constant frequency of 1 Hz. The response of the sample to these conditions was evaluated by the loss (G″) and storage (G′) moduli. All subsequent measurements, namely frequency sweep for the determination of G′ and G″, were performed at a frequency of 0.1 Hz to 100 Hz, with a shear strain of 0.05% and 10 samples per decade.

#### 2.2.5. In Vitro Release Testing (IVRT)

Experiments were carried out according to OECD “Guidance document for the conduct of skin absorption studies” [14]. The release of Clotrimazole was determined using static vertical Franz diffusion cells with a diffusion area of 1 cm^2^ and a receptor compartment of 3 mL. The membranes (Tuffryn^®^ membranes, Pall Europe, Hampshire, UK) were washed and equilibrated with ethanol/water (1:1) and then placed between donor and receptor compartments. The receptor medium, a mixture of ethanol/water (1:1), was appropriately screened based on Clotrimazole solubility studies to ensure the sink conditions during the experiment. The release media was continuously stirred with a magnetic bar and maintained at (35 ± 2) °C by a thermostatic water pump, assuring a temperature of 32 °C at the membrane surface (to mimic skin conditions).

All tests were conducted for 12 h. In total, about (0.3 ± 0.1) g of each formulation was evenly spread over the membrane surface. The donor compartment and the receptor sampling arm were enclosed with Parafilm^®^ to prevent evaporation. Aliquots of the receptor phase (200 μL) were withdrawn after 1, 2, 4, 6, 8, and 12 h, and analysed via HPLC (Section 2.2.7). After sampling, the same volume was replaced with fresh receptor phase maintained at (35 ± 2) °C.

The percentage of Clotrimazole released into the medium was calculated using the following Equation (3) [15]:

(3) $$\mathrm{Cumulative}\;\mathrm{release}\;\mathrm{percentage}=\sum_{t=0}^{t}\frac{M_{t}}{M_{0}}\times 100$$

where *M_t_* is the cumulative amount of Clotrimazole released at each sampling point, *t* is time, and *M_0_* is the initial weight of the Clotrimazole in the formulations.

The data obtained from IVRT were computed using DDSolver, an Excel-plugin module, and different kinetic models were evaluated for data fitting (Supplementary Table S3) [16].

In all models, F is the fraction (%) of drug released in time (*t*). The coefficient of determination (R^2^) was calculated for each model and used as an indicator of the goodness of fit to the data. The selection of a suitable model for fitting dissolution data is essential, not only for the quantitative evaluation of drug release characteristics, but also for comparison of dissolution profiles using model-dependent approaches.

The dissolution efficiency (DE) was calculated from the area under the dissolution curve up to a certain time (12 h), according to the following Equation (4):

(4) $$\mathrm{DE}=\frac{\int_{0}^{t}y\times dt}{y_{100}\times t}\times 100$$

where *y* is the percentage of the dissolved drug at time *t*.

#### 2.2.6. In Vitro Permeation Testing

In vitro permeation tests (IVPT) were performed for all formulations in Franz cells of static flow, using newborn pig skin obtained from a local slaughterhouse with a diffusion area of 1 cm^2^. The epidermis was visually inspected for any defects and then cut into sections, large enough to fit the Franz cells. The skin was then placed between the donor and receiver compartments of the cells. The experimental setup was the same as described for the in vitro release studies except the sample collection times which, in this permeation experiment were 2, 4, 6, 8, 12, and 24 h. Drug quantification was performed the same way as in drug release studies, according to the method described in Section 2.2.7.

The cumulative amount of permeated drug (*Qt*) through excised newborn pig skin was plotted as function of time and determined based on the following Equation (5):

(5) $$Qt=\frac{Vr\times Ct+\sum_{t=0}^{t-1}Vs\times Ci}{S}$$

where, *Ct* is the drug concentration of the receiver solution at each sampling time, *Ci* is the drug concentration of the sample applied on the donor compartment, and *Vr* and *Vs* are the volumes of the receiver solution and the sample, respectively. S represents the skin surface area (1 cm^2^).

According to Fick’s first law of diffusion, the steady-state flux (*J_ss_*, μg/cm^2^/h) can be expressed by [15], Equation (6):

(6) $$J_{ss}=DC_{0}P/h=C_{0}K_{p}$$

where *D* is the diffusion coefficient of the drug in the *stratum corneum*, *C_0_* represents the drug concentration in the donor compartment, *P* is the partition coefficient between the vehicle and the skin, *h* is the diffusional path length, and *K_p_* stands for the permeability coefficient.

The flux and *K_p_* of the formulations were measured and compared accordingly. The enhancement ratio (ER) for flux was calculated as the ratio between the flux of different formulations and the target flux value. The *J_ss_* and *K_p_* of the yielded formulations were calculated and compared. The permeation lag time, a parameter related to the required time to achieve the steady-state flux of a drug through the skin, was also considered for analysis.

#### 2.2.7. In Vitro Retention Testing

After the 24 h of the permeation study, the skin was carefully cleaned and cut into small pieces. Tetrahydrofuran (1 mL) was then added to the skin pieces, which were stirred for 1 min in a vertical mixer (29,000 rpm) and sonicated for 20 min to lyse the cells. After filtration (0.45 μm), the supernatant of this assay and samples of the receptor phase were analysed by HPLC, following the procedure described below.

#### 2.2.8. Drug Quantification

The content of Clotrimazole in the emulsion formulations was determined by HPLC, using a Hitachi Elite Chromaster (Hitachi, Tokyo, Japan) equipped with a pump, an autosampler, a column oven and a UV Detector. An analytical reversed-phase column Zorbax Eclipse XDB-C18, 5 μm, 4.6 × 150 mm (Agilent Technologies, Santa Clara, CA, USA) was used. An isocratic method was used, with a mobile phase with 60% (*v/v*) potassium dihydrogen phosphate and tetrabutylammonium hydrogen sulfate, and 40% (*v/v*) acetonitrile. A flow rate of 1.0 mL/min was used with a sample injection volume of 10 μL. The autosampler was maintained at 16 °C and the eluted peaks were monitored at 254 nm. The running time was 15 min. Chromatographic data was acquired and processed with the Empower 3^®^ Software.

#### 2.2.9. Statistical Analysis for Optimal Working Conditions

The results were expressed as mean ± standard deviation (mean ± S.D.) from three independent experiments, except otherwise specified. For the optimization studies, the MODDE^®^ software was used, and the model was considered valid when the p-value was lower than 0.05 (*p* < 0.05).

## 3. Results and Discussion

To obtain approval for a generic drug, several requirements must be met to prove equivalence to the RF. To this end, a guideline on quality and equivalence of topical products was followed, providing guidance on the quality, effectiveness and safety of a generic product. This guideline demands several products characterizations tests. A detailed product characterization facilitates life-cycle management and, where applicable, supports a claim of equivalence to the product being compared [5]. The results of this characterization are presented in the following sections.

### 3.1. Description and Composition of the Drug Formulation

The cream consists of Clotrimazole and the following excipients: sorbitan stearate, polysorbate 60, cetyl palmitate, octyldodecanol, cetostearyl alcohol at 10% (*w*/*w*), benzyl alcohol at 2% (*w*/*w*) and water as solvent. Clotrimazole is present at a concentration of 2% (*w*/*w*), with a maximum dose of once daily for three days (one dose is approximately 5 g of cream). The primary packaging for this medicine is an aluminum tube with a plastic cap and the secondary packaging is cardboard.

### 3.2. Identification of QTPP and CQAs

The QTPP should consider patient acceptability, ease of removal from the container and administration, bulk aesthetic properties such as appearance, spreadability, feel and the microstructure/physical properties. These elements need to be characterized and, when necessary, controlled as CQAs [5]. The characterization of the RF, its packaging and labelling are essential to define the QTPP of the generic drug product. Having the same components (Q1), in the same concentration (Q2), with the same microstructure (Q3), the reference is the most rational approach to formulate a generic dermatological product. CQAs are essential attributes that need to be closely monitored to assure that the microstructure of Q3 is similar to the reference [17].

In this work, the QTPP should be a white homogeneous oil-in-water emulsion for topical administration with a 2% (*w*/*w*) Clotrimazole concentration and with a characteristic odor. The identified CQAs were the assay (95.0–105.0% of the label claim), rheological behaviour (viscosity and oscillation), and product performance (in vitro release and permeation studies).

To establish the best experimental conditions for the optimal product performance, it is desirable to understand the effect of formulation and process variability on cream CQAs [18]. To this end, a multivariate optimization strategy was applied herein.

#### 3.2.1. Definition of Variables

First, we aimed at identifying the most critical parameters that influence the quality of the final formulation, using an Ishikawa diagram (Supplementary Figure S1) [15]. Using this approach, the main problems identified were the rheological behaviour, drug release and drug permeation of the obtained formulations not being similar to the reference. Then, we aimed at identifying the excipients that may lead to these problems. Based on the bibliographic review carried out on the properties of each excipient, two of them were selected as possible critical parameters in the formulation: cetyl palmitate and octyldodecanol. The content variation of these two excipients was selected based on the literature review and several creams with different amounts of these compounds were formulated and characterized to evaluate their impact on the formulations [19,20,21]. The following model responses were selected, as they are important variables when comparing the generic drug with the reference drug, such as apparent viscosity, loss modulus (G′), storage modulus (G″), drug release after 12 h, DE after 12 h, drug permeation, and retention after 24 h.

#### 3.2.2. Definition and Application of the Experimental Design

The qualitative and quantitative formulation of the generic drug was used as a starting point for optimization. The experimental goal of this design is to optimize the formulation of the generic drug and, for that purpose, it was decided to apply a Central Composite Orthogonal design. Considering the criteria previously defined, MODDE^®®^ proposed a set of experiments to study the formulation of the generic drug. To evaluate the effect of those variables on cream CQAs, DoE formulations were characterized for the main quality attributes.

### 3.3. Product Characterization

The microstructure and physical characterization of the formulations should include pH, rheological profile, polymorphism, API, and droplet size determination, together with a detailed understanding of both release kinetics and permeation behaviour [22].

#### 3.3.1. Macroscopic Appearance and pH Determination

All the emulsions presented a homogeneous appearance, with a bright white color and a characteristic odor. No phase separation was observed in the centrifugation tests. The pH values of the DoE formulations varied between 5.8 and 7.6 (Supplementary Table S4). These values were not considered in the experimental design since no significant variations were detected, and the pH values could be adjusted at the end of the formulation manufacturing.

Some studies have shown that the Clotrimazole is a weak base with pKa = 6.12, is stable in the pH range of 1.2–7.5, but it degrades in strongly acidic and basic media. However, it is reported to be practically insoluble in water [23,24]. Thus, it is important to identify suitable oil, surfactant/cosurfactant systems providing maximum solubilization of the drug under investigation, to achieve optimum drug loading. Clotrimazole exhibited higher solubility in synthetic oils such as octyldodecanol [24], which are less affected by changes in pH and ionic strength. These characteristics are important for the successful development of a Clotrimazole emulsion in order to achieve pH-independent solubility. Thus, the different pH values are due to the different concentrations of octyldodecanol present the in DoE formulations, and consequently different degrees of solubilization of Clotrimazole. These results were confirmed by microscopy analysis (results not shown). In addition, the pH of the skin is normally acidic, ranging between 4 and 6 [25]. The acquired results are slightly above the skin pH range but are stated that topical formulations pH between 5 and 7 do not seem to cause skin irritation, suggesting a safe application of the DoE formulations [26].

#### 3.3.2. Droplet Size Distribution

Droplet size analysis was carried out to detect possible changes in the distribution of the overall size and alterations in the volume mean diameter, and the results are summarized in Supplementary Figure S2.

The results show that the formulations with the lowest amount of octyldodecanol (F1, F2, and F7) have the lowest percentage of smaller droplets, with a diameter range of 1 to 10 μm, suggesting that octyldodecanol increases the droplet size. Formulations F2 and F4, which have the highest amounts of cetyl palmitate, also have a low percentage of 10 μm droplets. However, this result was not observed for F6, the other formulation with the highest amount of cetyl palmitate (F6). This suggests that the droplet size is not only influenced by ingredients, but also by external factors such as homogenization speed and/or time, the cooling rate, the used equipment, the shelf-life, among others. It has been previously shown that the emulsion droplet sizes are prone to change during the shelf-life of the product [27].

#### 3.3.3. Rheological Characterization—Structure Analysis

Rheology can be used as a tool to assess parameters that help in the evaluation of the release of active compounds from vehicles [28]. The rheological behaviour of pharmaceutical semisolid preparations significantly affects the manufacturing process, appearance, administration, stability, homogeneity of the incorporated drug, accuracy of dosing, adhesion to the site of application, drug release, and resulting therapeutic effect of the product [29]. Thus, for semisolid pharmaceutical dosage forms such as creams, it is fundamental to fully understand the rheology of the product, as this may affect both its application and delivery of the drug to and across the skin (therapeutic effectiveness) [30]. To ensure that the defined rheological properties were fulfilled, suitable parameters were determined.

##### Rotational Measurements

Viscosity is an important factor for semisolid formulations as it may influence the release of drug by altering the diffusion rate from the vehicles. The viscosity profile of a formulation provides important information about its production, processing, and performance. Viscosity is considered an indicator of the stability of a product and is correlated with internal structure robustness [27].

**Figure 2:**
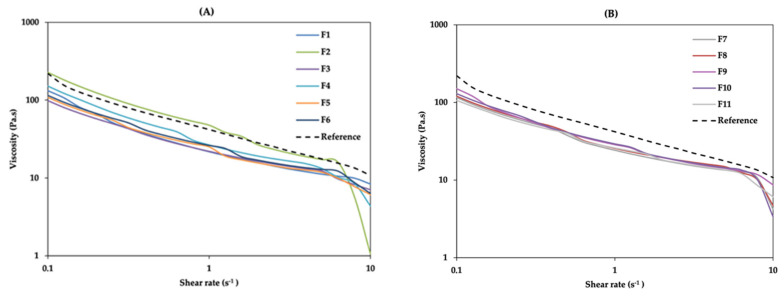
Effect of independent variables on cream viscosity: (**A**) F1–F6 and RF; (**B**) F7–F11 and RF (reference formulation). The results shown are means, *n* = 3.

Figure 2 shows the obtained results from the viscosity of the DoE and RF. It can be observed that the formulations with lower concentration of cetyl palmitate (F1, F3 and F5) have lower viscosity profiles, although not considerably different from the other formulations. However, some formulations loose considerable viscosity when a 10 s^−1^ shear rate is reached. This is the case of F2, which, despite containing 4% cetyl palmitate, has a low concentration of octyldodecanol (5%). Cetyl palmitate and octyldodecanol, as well as their interaction, were considered critical parameters, since both excipients have an impact on the formulation’s viscosity. Previous studies, using different concentrations of different emollients, showed that the amount of this excipient significantly influences the viscosity and spreadability of a cream [31]. Viscosity is highly dependent on cetyl palmitate since this acts as a viscosant, i.e., it increases the viscosity of the formulation.

The highest viscosity was observed for the formulations with smaller droplet size. Considering the impact of the independent variables (Figure 2 and Supplementary Table S5), higher values of cetyl palmitate resulted in increased viscosity. For a complete flow behaviour characterization of all formulations, different mathematical models (Supplementary Table S2) were fitted to the experimental data. Rheological curve fitting allows confidence intervals to be set around the standard regression coefficients, instead of comparing the standard curves with a specific flow, and thus it can be decided whether the material tested is within or outside the specifications of the model in question [32,33].

Supplementary Tables S6 and S7 show the fitting parameters of the models described in Table S2 to the measured data. Among the five flow models considered in this work, the Herschel-Bulkley was the best for predicting the flow behavior of DoE formulations. This model is an extended version of a simple power-law flow equation to include a yield stress term and is very useful to quantitatively describe the steady shear flow behavior of several types of soft materials. The obtained results suggest that all formulations are shear-thinning according to the Herschel–Bulkley model, because they have a viscosity value lower than or equal to 1. Since the emulsions behave as a shear-thinning fluid, they are suitable for topical administration.

The testing of pharmaceutical semisolids by an oscillatory method provides a wealth of fundamental rheological information [34]. The tests for analysis of viscoelastic materials are designed in order not to destroy the structure, so that measurements can provide information on the intermolecular and interparticle forces in the material [19].

The storage modulus (G′) is a measure of the deformation energy stored by the sample during the shear process, representing the elastic behaviour of a test material. The loss modulus (G″) is a measure of the deformation energy used by the sample during the shear process, representing the viscous behavior of the material and, afterwards, it is lost for the sample [33]. This energy is spent during the process of changing the material’s structure. Energy losing materials show an irreversible deformation behaviour since their shape changes after a load cycle. Usually, for oil-in-water (O/W) creams, G′ > G′′, indicating that the elastic properties exceed the viscous ones [27]. The loss factor (Tan δ), defined as the ratio between G″ and G′, has also been used to model the structure in the system. A value of Tan δ > 1 indicates a liquid-like behaviour, whereas a value < 1 means solid-like behaviour. A low value of Tan δ often means that the formulation has a significant structure.

**Figure 3:**
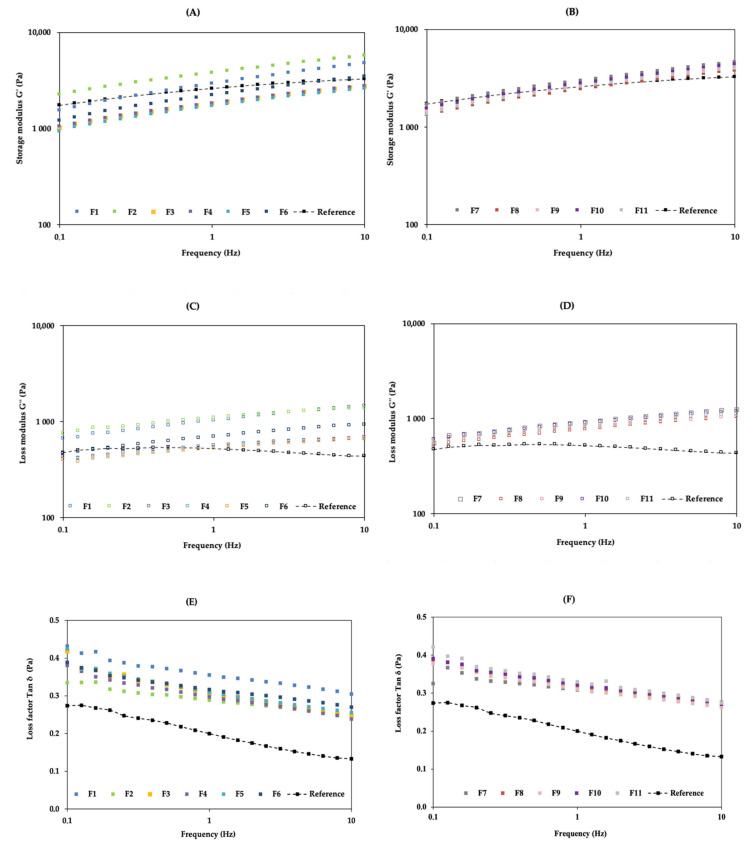
Effect of independent variables on formulations’ storage modulus (G′): (**A**) F1–F6 and RF; (**B**) F7–F11 and RF; effect of independent variables on formulations’ loss modulus (G″): (**C**) F1–F6 and RF; (**D**) F7–F11 and RF; effect of independent variables on formulations’ loss factor (Tan δ): (**E**) F1–F6 and RF; (**F**) F7–F11 and RF. The results shown are means, *n* = 3.

The results obtained for G′, G″, and Tan δ are shown in Figure 3. The lower the amount of octyldodecanol in the formulations (F1, F2, and F7), the higher the values of G′ and G″. The influence of the emollient on the spreadability and rheological behaviour of semisolid formulations has been reported in previous studies [35,36]. The results also show that, in the tested range (0.1–10 Hz), G′ is significantly higher than G″ for all DoE formulations, suggesting that the microstructure of the cream is highly organized and dominated by cohesive forces. This behaviour is typical of a viscoelastic liquid and can be described by a mechanical model made up from a combination of springs (elastic elements) and dashpots (viscous elements). At high frequency, the springs can elongate and contract under imposed shear, but the dashpots have very little time in which to move [33]. The smaller the ratio of G″/G′ (=Tan δ), the more rubbery or elastomeric the behaviour [34]. The Tan values were lower than 1 for all formulations, indicating a solid-like behaviour.

### 3.4. Product Performance

To test the performance of the developed formulations comparatively to the RF, it is important to establish their effectiveness in vitro. The in vitro drug release and permeation analysis may be suitable to test the sameness (Q3) of Q1/Q2 equivalent topical dermatological products concerning their performance [30].

#### 3.4.1. IVRT

The vehicles that are used to deliver topical therapies can considerably influence drug performance, affecting its delivery and physical appearance [37]. The purpose of the release studies was to study the release behaviour of the drug from the developed formulations, compare it with that of the RF and infer the therapeutic benefit of the product.

**Figure 4:**
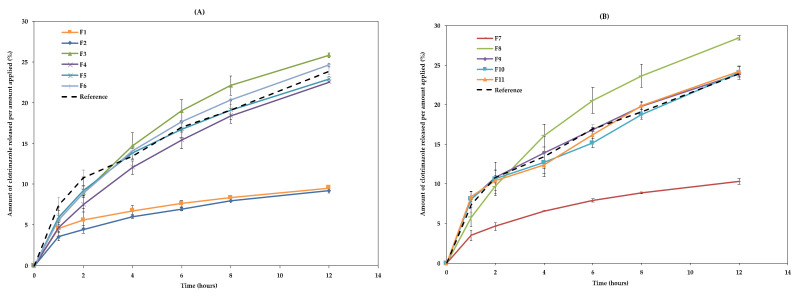
Cumulative release drug through membranes: (**A**) F1–F6 and RF; (**B**) F7–F11 and RF. The results are mean ± SD, *n* = 3.

The release of the drug from the different formulations was measured through a synthetic membrane. Figure 4 shows the cumulative released drug amount plotted against time, which was used to determine the release rate.

The results show that most formulations had a similar behaviour to the RF, although F1, F2, and F7 showed a significantly lower release. These formulations contain the lowest amounts of octyldodecanol, 5% (F1 and F2) and 4.3% (F7). On the other hand, formulations F3 and F8, containing the highest amounts of octyldodecanol (15.0% and 15.7%, respectively), had a slightly higher release than the others. Taken together, these results suggest that the excipient with the greatest impact on the release of the drug is octyldodecanol (Figure 4), with release increasing with increasing concentration of this excipient in the formulation.

Previous studies have shown that emollients can differ greatly in their properties and their effects on the skin. These excipients can have a significant impact on the formulation’s release and skin absorption and also emphasize that the rheological characteristics of a formulation can affect the release of the drug. In this study, plastic viscosity was correlated with the diffusion of the drug and it was observed that the viscosity was inversely proportional to the release rate [38].

Supplementary Table S8 summarizes the released drug amounts after 12 h, the DE and the area under the curve (AUC). As expected, formulations that contain a lower amount of octyldodecanol have a lower DE and AUC.

Three different kinetic models were fitted to the data obtained in the in vitro release studies (Supplementary Table S9). For most formulations, the best fitting was obtained with the Korsmeyer–Peppas model. This model ($F=k_{KP}\cdot t^{n}$) allows the characterization of the different release mechanisms through evaluation of the diffusion release exponent (n) [39]. The n values obtained for formulations F1, F2, F7, F9, F10, F11, and RF suggest that these formulations have a Fickian behaviour. Fickian diffusional release occurs via usual molecular diffusion of the drug due to a chemical potential gradient [40]. On the contrary, the n values obtained for formulations F3, F4, F5, F6, and F8, suggest a non-Fickian behaviour. Taken together, these results indicate that the increase in the amount of octyldodecanol leads to a non-Fickian behaviour. The value of k represents the release rate constant of the respective kinetic models. In this case, the formulations with the highest k values and also the most similar to the RF were the central points (F9, F10 and F11), meaning these have the fastest release through the artificial membrane.

The similarity factor (f2) between DoE formulations and RF was also obtained using DDSolver. The US Food and Drug Administration (FDA) and the EMA suggest that two dissolution profiles are considered similar when 50 < f2 < 100% [31]. The obtained results of the similarity between DoE formulations and RF ranged between 53.8% (F1) and 98.2% (F9). Thus, according to the FDA, all formulations are similar to the RF.

#### 3.4.2. IVPT

The permeation profile was assessed through newborn pig skin. In order to unravel the amount of Clotrimazole permeated, the receptor phase was analysed by HPLC as described in Section 2.2.7.

**Figure 5:**
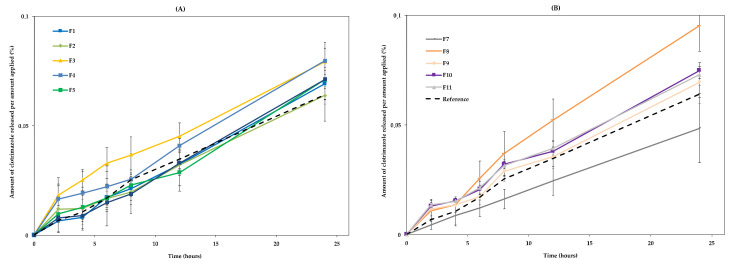
Drug permeation profile through newborn pig skin: (**A**) F1–F6 and RF; (**B**) F7–F11 and RF. The results are mean ± SD, *n* = 3.

Such as in release studies, the formulations with higher amounts of octyldodecanol (10% and 15%) showed the highest permeation profiles (F3, F4, and F8) (Figure 5). On the contrary, the formulation containing the lowest amount of octyldodecanol (F7), showed the lowest permeation profile, although the differences from the RF were not statistically significant. These results suggest that the presence of octyldodecanol contributed to the percutaneous penetration of the drug. Considering that Clotrimazole is enough solubilized in the formulation to ensure vehicle–skin interface saturation, the greatest permeation parameters result from the vehicle–skin interactions, rather than Clotrimazole–skin interactions. The results also suggest that the developed creams are safe for human topical application, since only a small percentage of the applied drug is permeated through the epidermis during 24 h.

Studies by other authors also showed that the addition of octyldodecanol to the formulation increases the permeation rate [41,42]. Emollients are agents that keep the moisture intact on the skin, keeping it soft. This moisture keeps the skin hydrated for a longer period, increasing the water content by occlusion, thereby loosening the intercellular lipid layer and enhancing the permeation of drug molecules [42,43].

The fluxes (J_ss_), permeability coefficients (K_p_) and lag times were obtained upon fitting the single curves of the permeation profiles in the linear plot region (between 4 and 24 h). According to the results shown in Table 1, the formulation with the highest concentration of octyldodecanol was the one that presented the highest flow (F8). The ER gives an insight as to whether the formulation under study exhibits a greater or lesser flow compared to the RF. Q_12 h_ and Q_24 h_ represent the amount of permeated drug (μg/cm^2^) after 12 and 24 h, respectively.

Previous studies emphasized that the composition of the emollient and, more specifically, the emollient content, highly influences the penetration and absorption in the skin and helps to understand the spreading behaviour [38].

**Figure 6:**
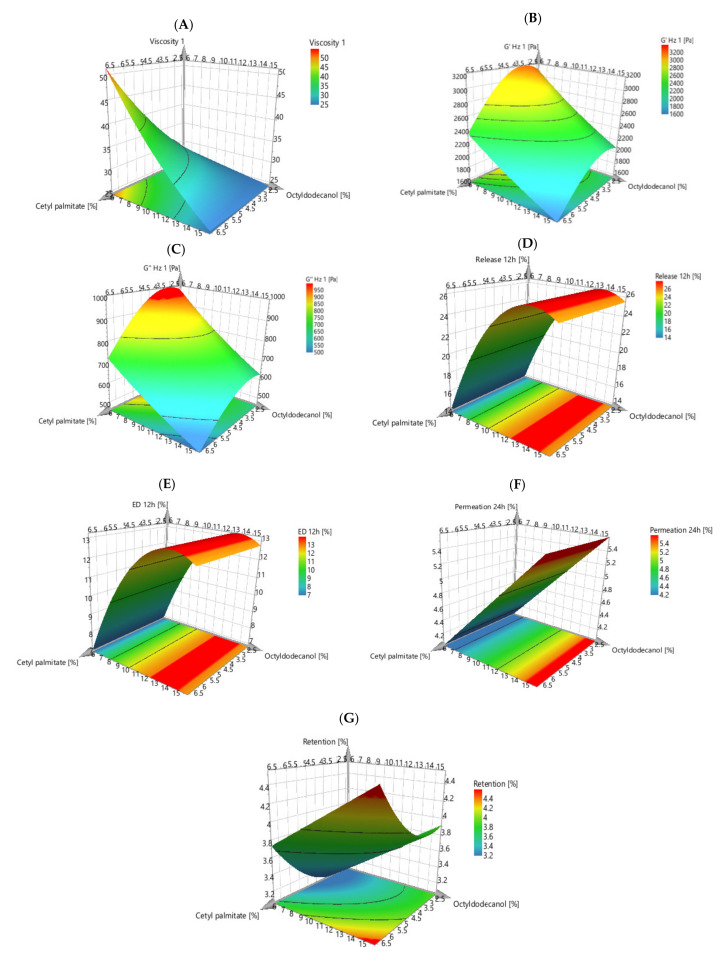
Response surface plots of the fitted model for viscosity (**A**), storage modulus (**B**), loss modulus (**C**), in vitro release studies (**D**), dissolution efficiency (**E**), in vitro permeation studies (**F**) and retention studies (**G**).

Following the in vitro permeation studies the retention study aimed to quantify the drug that was retained in the skin after 24 h. The obtained values of DoE formulations ranged between 2.7 ± 0.1 μg/cm^2^ (F7) and 4.3 ± 0.6 μg/cm^2^ (F4), which are similar to the one obtained for the RF (3.3 ± 0.6 μg/cm^2^). Figure 6 shows the response surface plot for this study.

### 3.5. Optimal Working Conditions

Design space is a multidimensional combination and interaction between the independent variables that assure quality. Working within the DS is not considered a change, since different experimental conditions may produce the same qualified product [22,44].

To understand how the factors influence the response, a multiple linear regression (MLR) model was used, whereby the response is estimated from the factors to evaluate the respective impact, assuming that there is a linear relationship between them [45]. Analysis of variance (ANOVA, Supplementary Table S10) was used to evaluate the model’s performance, by quantifying the variability in two ways: (i) test of significance of linear regression, in which the model is satisfied when the probability value (prob) is less than 0.05; (ii) the lack of fit test where a prob > F > 0.05 suggests that the fitted models demonstrated a great ability in predicting the responses [46].

The coefficient list (Supplementary Table S11) displays the scaled and centered coefficients for the selected responses and the significance levels. These coefficients indicate the influence of different factors and their interactions in the variation of responses [46]. *p*-values associated with non-significant coefficients at the selected confidence level are shown in green.

An optimization study was performed to identify and establish the impact of cetyl palmitate and octyldodecanol and their interactions on predefined CQAs. The response surface methodology was applied to establish the DS (Figure 6). The factors that were shown to affect the formulation quality were used to create the DS. The green space corresponds to a range of combinations for which the responses remain within the pre-defined acceptable limits. To obtain a region of values that could be explored with considerable answers, a DS plot was made, and an optimal region was established. By compiling all the results, the software provided the ideal quantities for a formulation as similar as possible to the RF. These values were obtained with a failure probability of 0% excluding the model error. Thus, the software provided the setpoint that helps to define the acceptable value for all individual factors to ensure that all CQAs are fulfilled and to reach the QTPP. A final formulation was manufactured with the provided quantities.

### 3.6. Final Formulation

The criteria for the selection of the desired setting of independent variables (cetyl palmitate and octyldodecanol) were mainly based on the highest possible response values. To get the desired response, a final formulation (FF) was prepared using the optimal values proposed by the DoE. The FF was characterized and evaluated by comparison with the RF.

#### 3.6.1. Droplet Size Distribution

Experimental measurements of droplet size show a bimodal population with different profiles for the FF and the RF (Supplementary Figure S3). The droplet size distribution values are summarized in Table 2, and show that the RF has lower droplet sizes, below the d(10) percentile, while the FF has slightly bigger droplets, reaching d(90).

An increase in the speed of rotation of the agitator at different scales potentiates the phenomenon of droplet breaking, which leads to the formation of smaller emulsion droplets. A high mixing rate favors the formation of smaller droplets, as it is directly related to the average shear rate created in the manufacturing of the sample, which is responsible for the deformation and breakage of droplets into smaller ones [47,48]. The differences in droplet size may be due to the different production scales of the formulations being compared: the FF was produced on a laboratory scale and the RF was produced on an industrial scale. It is expected that when the FF is produced at an industrial scale, these parameters will be similar to those of the RF.

#### 3.6.2. Rheological Characterization

In terms of formulation flow, apparent viscosity and thixotropic behaviour were considered. Rotational shear experiments were conducted to measure the ability of each system to resist structural deformation during the standardized shearing procedure.

##### Rotational Measurements

**Figure 7:**
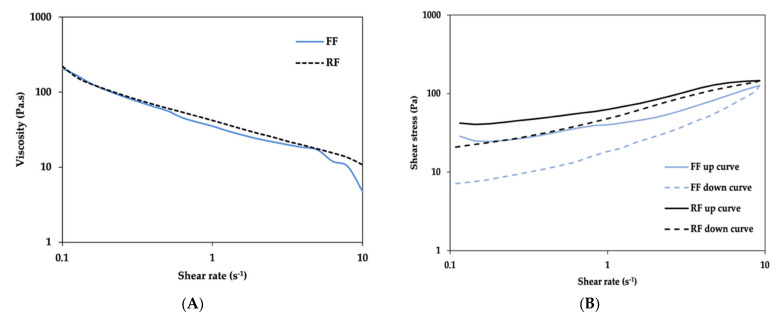
Viscosity profile of the FF and RF (**A**) and Flow curves represented by shear stress as a function of shear rate for the FF and RF (**B**). Data shown are means, *n* = 3.

The viscosity curves obtained for the formulations are shown in Figure 7A. The FF and RF have similar viscosity profiles, with apparent viscosities values at a shear rate of 1 s^−1^ of 35.1 Pa.s for the FF and 41.9 Pa.s for the RF.

The apparent viscosity of both formulations decreased with increasing shear rate and exhibited non-Newtonian behaviour, which confirms their shear-thinning character. To develop a suitable semisolid formulation for topical administration of drugs this property should be characteristic of the system [49]. Thixotropy is a reversible phenomenon exhibited by non-Newtonian materials, characterized by a reduction in the apparent viscosity when the material is subjected to a constant shear rate (ascendant curve; structure deformation), returning to its original viscosity when the shear forces decreased (descendent curve; structure recovery) [8]. This study provided qualitative information about the formulation breakdown during a deformation and recovery cycle. During extrusion from the container, the emulsion formulation undergoes repeated shear forces. To prevent structural breakdown and ensure stability during use, the formulation structure recovery must be ensured through a thixotropic behaviour, making this a good stability indicator. The flow curves shown in Figure 7B suggest that FF and RF are thixotropic systems, since hysteresis loop areas were promptly observed with the up curves located above the down curves, which are not completely recovered when the shear rate is ceased. As can also be observed in the same figure, the RF needs higher shear stress per unit of area to be disrupted and flow than the FF, an indication that the RF is more structured than FF, which is expected since RF is produced at an industrial scale. Thus, it is also expected that when FF is produced at this scale, it will have smaller and more homogeneous droplet size, which will increase its viscosity [47].

It can also be observed that the RF has a better recuperation than the FF and consequently a smaller hysteresis area (375.5 ± 79.7 cm^2^ for FF and 270.2 ± 38.1 cm^2^ for RF). This could influence the release rate of drug, owing to the time needed for the formulation to recover its viscosity and initial structure, when the imposed shear rate decreases. Thus, active substances incorporated in more thixotropic systems have more time to be released faster in the period of low viscosity caused by the spreading of the emulsion over the skin [50]. However, and as previously referred, these differences should fade when the FF is produced at an industrial scale.

Supplementary Table S12 summarizes the flow models (Bingham, Casson, Cross, Herschel–Bulkley, Power law, and Sisko) adopted in this study and their flow characteristics. The consistency index (k) in Binghan, Cross, Herschel–Bulkley, Power law, and Sisko models is a measure of the viscous nature of an emulsion, with a higher k value reflecting a stronger emulsion network. The FF had a lower k value on most models indicating a low viscosity value, while the RF had a higher k value, indicative of a stronger network [40].

Yield stress affects the spreading behaviour of the cream and plays an important role in the physical stability of the product. There is no generally accepted standard procedure to determine a yield stress value. One of the most common techniques is an indirect measurement which involves an extrapolation of the shear stress-shear rate data obtained from conventional rheometers, using a rheological model [29]. Using the model that had the best fitting, the obtained yield stress results were quite concordant. The results obtained after model fitting show that both FF and RF are shear-thinning, since they have a viscosity value less than or equal to 1. As the emulsions behave as a shear-thinning fluid, they are suitable for topical administration.

A high correlation coefficient, which measures the proportion of the total variation in the mean explained by the regression, was obtained in most of the fitted models. Herschel-Bulkley is a good and complete fitted model that describes the steady shear flow behavior of several types of soft materials [28]. The Sisko model is also suitable but with less accuracy.

##### Oscillatory Measurements

The results displayed in Supplementary Figure S4 show the variation of G′ and G″ with frequency (Hz). For both formulations, G′ tends to increase with increasing frequencies. This behaviour is typical of a viscoelastic formulation, suggesting that the microstructure of the cream is highly organized and dominated by cohesive forces. When G′ > G″ for a viscoelastic material, the shear energy is temporarily stored during the test and can be retrieved later as usually occurs in O/W emulsion systems. Creams with this feature usually exhibit high stability [28]. Despite the increase in G″, both formulations presented a higher value of G′, maintaining the solid-like properties. The FF and RF presented similar values of G′, G″ and Tan δ at 1 Hz (3095.0 and 2611.3 Pa for G′; 994.3 and 522.9 Pa for G″; 0.32 and 0.20 Pa for Tan δ, respectively.

#### 3.6.3. Product Performance (IVRT and IVPT)

**Figure 8:**
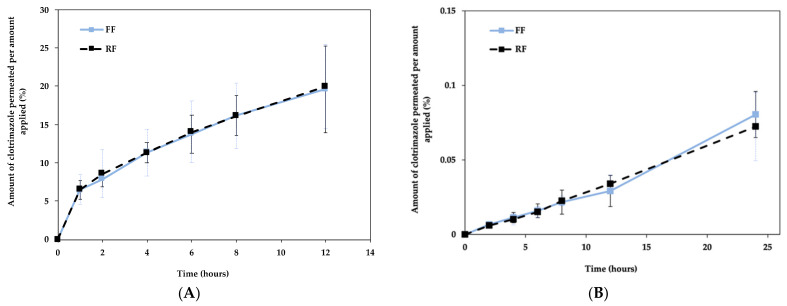
Release profile of drug from the FF and RF through synthetic membranes (**A**) and permeation profile of drug from the FF and RF through newborn pig skin (**B**). Data are mean ± SD, *n* = 6.

The release rate of a drug is dependent on its solubility and viscosity since it can limit the diffusion rate from the vehicles and, consequently, the drug available for skin permeation. The amount of drug released through the synthetic membrane is displayed in Figure 8A, which shows the cumulative released drug amount plotted against time for the release rate determination. Both formulations show a quick release in the first hour, gradually increasing until 24 h, with very similar release profiles. The cumulative % of drug released was approximately 20% for both formulations.

According to the Stokes law, the diffusion coefficient of the drug is inversely proportional to viscosity, since more viscous formulations will retain the active substance, hindering its release from the vehicle for longer [26]. Bearing in mind that the viscosity results are quite similar for FF and RF, the drug release results are also similar.

The permeation profiles of the FF and RF through newborn pig skin were assessed, and Figure 8B shows the cumulative permeated drug amount plotted against time.

According to the results obtained in the DoE in the previous section, the excipient with the greatest impact on the release of the drug was octyldodecanol. Considering the similar results of drug release for both formulations (Table 3), this supports that the concentration of this excipient, suggested by the software, was suitable. Differences observed concerning the droplet size, the thixotropic behavior, and the frequency sweep did not affect the permeation results, suggesting that although the physical properties of the cream are very important for the effectiveness of the final product, the choice of the concentration of some raw materials can have a high impact on the penetration/retention of the drug into the site of action and consequently on its availability [26]. Three different kinetic models were fitted to the in vitro release data and the parameters obtained are shown in Supplementary Table S13. The adjusted coefficients of determination (R^2^_adjusted_) and AIC (Akaike information criterion) estimated for each model were used as an assessment of the goodness-of-fit and the model ability to describe a given dataset. When comparing several competing models, the best fitting is the one with the maximum R^2^_adjusted_ value and minimum AIC value [51].

For both formulations, the best fitting was obtained with the Korsmeyer–Peppas model (R^2^_adjusted_ = 0.91 for FF and R^2^_adjusted_ = 0.99 for RF) and the parameters obtained suggest a Fickian behaviour for both formulations. Comparing AIC values, the Korsmeyer–Peppas model also presented lower values for both formulations, which corroborates the R^2^_adjusted_ results.

The similarity factor (f2) between the FF and RF, obtained with DDSolver, was 98.8%, which supports that the two formulations have similar profiles.

The permeation profiles for FF and RF are similar, suggesting that the formulations are equivalent. Table 3 summarizes the parameters of the drug permeation measurements after 12 and 24 h. At different factor level combinations, similar values were obtained for flux, permeability coefficients (K_p_) and lag time, upon fitting the single curve of the permeation profile in the linear region. At the end of the in vitro permeation studies, the newborn pig skin was removed from the Franz cells and treated as described in Section 2.2.6, in order to quantify the drug that was retained in the skin after 24 h. Also in this case, the retention percentages obtained for the FF and the RF are very similar.

Considering the results obtained in all the tests, the differences noted in the droplet size, thixotropic behavior, and frequency sweep did not significantly affect the performance of the formulation with respect to the in vitro release and permeation of the drug.

## 4. Conclusions

Demonstrating the equivalence of a new topical drug to an existing drug is certainly important to ensure its safety, quality and effectiveness. In the present work, a whole path was followed along the guideline on quality and equivalence of topical products in which the first step was demonstrating the quality of the product and then the equivalence. Even though a 2%(*w*/*w*) Clotrimazole cream was used as a case study, the same rationale can be transposed to other semisolid products. Thus, a comprehensive structure for the development of a generic product is presented in order to obtain a product with quality and with the desired therapeutic action. In light of the new regulatory requirements, the importance of a detailed microstructure and physical characterization of topical semisolid dosage forms is undeniable.

Cetyl palmitate and octyldodecanol amounts are critical material attributes, due to their significant impact on formulation rheological properties. The results obtained herein showed that low concentration of cetyl palmitate led to less viscous formulations, while the concentration of octyldodecanol had impact on the cream structure (G′ and G″). Regarding in vitro studies, the octyldodecanol had an influence on the release, permeation and retention of the active substance, having a dominant role in dermatological therapy. Thus, octyldodecanol significantly contributes to drug-vehicle-skin interaction. These results show that in order to guarantee the stability, availability of the active substance, and consequently the effectiveness of a semisolid formulation, it is important to choose an appropriate system. QbD is a particularly useful tool in formula optimization. Considering all the results, MODDE^®®^ analysed and successfully obtained an answer for the ideal quantities of the two factors considering the intended target. The analysis of the FF against RF revealed similarities in several of the analysed parameters, particularly the in vitro performance, suggesting that this formulation is a promising generic and can advance to scale up.

## Figures and Tables

**Table 1:** Permeation parameters according to experimental permeation data of DoE formulations and RF. Data are mean ± SD, *n* = 3.

Samples	J_ss_ (μg/cm^2^/h)	ER (J_ss_)	K_p_ (cm/h)	Q_12 h_ (μg/cm^2^)	Q_24 h_ (μg/cm^2^)	Lag Time (h)
F1	0.20 ± 0.03	1.16	9.9 × 10^−6^ ± 1.7 × 10^−6^	2.2 ± 0.1	4.7 ± 0.2	2.6 ± 0.6
F2	0.17 ± 0.02	0.97	8.4 × 10^−6^ ± 1.1 × 10^−6^	2.1 ± 0.5	4.2 ± 0.3	3.1 ± 1.8
F3	0.18 ± 0.05	1.06	9.2 × 10^−6^ ± 2.3 × 10^−6^	3.1 ± 0.1	5.5 ± 0.7	6.4 ± 5.0
F4	0.19 ± 0.01	1.14	9.9 × 10^−6^ ± 5.5 × 10^7^	2.6 ± 0.3	5.0 ± 0.5	2.9 ± 1.1
F5	0.20 ± 0.03	1.16	1.0 × 10^−5^ ± 1.4 × 10^−6^	1.9 ± 0.5	4.9 ± 0.6	1.9 ± 0.6
F6	0.16 ± 0.02	1.13	9.7 × 10^−6^ ± 7.3 × 10^−7^	2.1 ± 0.4	4.4 ± 0.2	1.4 ± 0.6
F7	0.13 ± 0.03	0.73	6.4 × 10^−6^ ± 1.7±10^−6^	1.6 ± 0.3	3.1 ± 0.9	0.4 ± 0.0
F8	0.26 ± 0.00	1.50	1.3 × 10^−5^ ± 2.2 × 10^7^	3.4 ± 0.4	6.2 ± 0.4	1.1 ± 0.4
F9	0.18 ± 0.01	1.03	8.9 × 10^−6^ ± 6.5 × 10^−7^	2.3 ± 0.2	4.4 ± 0.3	0.9 ± 0.2
F10	0.17 ± 0.01	1.01	8.7 × 10^−6^ ± 4.8 × 10^−7^	2.4 ± 0.2	4.5 ± 0.3	1.9 ± 0.5
F11	0.18 ± 0.00	1.02	8.9 × 10^−6^ ± 1.4 × 10^−6^	2.5 ± 0.2	4.6 ± 0.2	1.9 ± 0.8
RF	0.17 ± 0.03	1.00	8.7 × 10^−6^ ± 1.2 × 10^−6^	2.3 ± 0.2	4.2 ± 0.3	1.7 ± 0.8

Jss—fluxes, ER—enhancement ratio, Kp–permeability coefficients, Q12 h—permeated amount of drug after 12 h, Q24 h—permeated amount of drug after 24 h.

**Table 2:** Droplet size distribution of FF and RF stored at room temperature. Data were obtained one week after production and are mean ± SD, *n* = 3.

Samples	Droplet Size Distribution (μm)			
	Span	d(10)	d(50)	d(90)
FF	2.9 ± 0.0	1.5 ± 0.1	9.2 ± 0.8	29.1 ± 2.7
RF	4.7 ± 1.1	1.2 ± 0.0	5.3 ± 0.6	20.9 ± 0.3

d—diameter values corresponding to percentiles of 10%, 50%, 90%.

**Table 3:** Values obtained from the in vitro release and permeation studies of the FF and RF after 12 h. Data are mean ± SD, *n* = 6.

Samples	Release _12h_ (%)	DE _12h_ (%)	AUC	J_ss_ (μg/cm^2^/h)	ER (J_ss_)	K_p_ (cm/h)	Q_12 h_ (μg/cm^2^)	Q_24 h_ (μg/cm^2^)	t_lag_ (h)	Retention (%)
FF	19.6 ± 5.6	9.8 ± 2.8	156.2 ± 25.7	0.22 ± 0.03	1.13	1.1 × 10^−5^ ± 1.7 × 10^−6^	1.9 ± 0.8	4.9 ± 0.7	1.7 ± 0.9	3.5 ± 0.9
RF	19.9 ± 5.5	9.9 ± 2.7	158.3 ± 44.8	0.20 ± 0.06	1.00	9.7 × 10^−6^ ± 2.8 × 10^−6^	2.2 ± 0.3	4.5 ± 0.9	1.7 ± 0.0	3.3 ± 0.6

Release _12h_—released amount of drug after 12 h, DE _12h_—dissolution efficiency after 12 h, AUC—area under the curve, Jss—fluxes, ER—enhancement ratio, Kp—permeability coefficients, Q_12 h_—permeated amount of drug after 12 h, Q_24 h_—permeated amount of drug after 24 h, t_lag_—lag time.

## Data Availability

Not applicable.

## Supplementary Materials

The following are available online at https://www.mdpi.com/article/10.3390/pharmaceutics13040555/s1, Figure S1: Ishikawa diagram to identify CQAs, Figure S2: Droplet size distribution of DoE formulations and RF at room temperature: (A) F1–F4 and RF; (B) F5–F8 and RF; (**C**) F9–F11 and RF. The results shown are means, *n* = 3, T Figure S3: Droplet size distribution of FF and RF stored at room temperature. The results shown are means, *n* = 3, Figure S4: Storage (G’) and loss (G’’) moduli of the FF and RF. Data shown are means, *n* = 3, Table S1: Experimental design conditions, design and experimental matrices according to Central Composite Orthogonal (CCO) design., Table S2: Mathematical models used in the fitting of rheological data, Table S3: Models used in DDSolver for fitting drug release data, Table S4: pH values of DoE (Design of Experiments) formulations and RF (reference formulation) at room temperature., Table S5: Comparison of the effect of independent variables on cream rheological profile. Data are expressed as mean ± SD, *n* = 3, Table S6: Regression parameters from Bingham, Casson and Cross models fitted to the rheological data. Data are expressed as mean ± SD, *n* = 3, Table S7: Regression parameters from Herschel-Bulkley, Power law and Sisko models fitted to the rheological data. Data are expressed as mean ± SD, *n* = 3, Table S8: Obtained values from *in* vitro release studies of DoE formulations and RF after 12 h. Data are expressed as mean ± SD, *n* = 3, Table S9: Regression coefficients obtained by fitting First-order, Higuchi and Korsmeyer-Peppas mathematical models to the release data from DoE formulations and RF after 12 h. Data are expressed as mean ± SD, *n* = 3, Table S10: Summary of ANOVA parameters concerning the fitted model’s characterization, Table S11: Summary of regression analysis results for measured responses, Table S12: Regression parameters from Bingham, Casson, Cross, Herschel-Bulkley, Power law and Siso models fitting to the rheological data. Data are expressed as mean ± SD, *n* = 3, Table S13: Regression coefficients obtained from the fitting of First-order, Higuchi and Korsmeyer-Peppas mathematical models to the release data from the FF and RF. Data are mean ± SD, *n* = 6.
