# Supplementary material for: Complying with the Guideline for Quality and Equivalence for Topical Semisolid Products: The Case of Clotrimazole Cream
Source: Pharmaceutics. 2021 Apr 14;13(4):555. doi: 10.3390/pharmaceutics13040555 (PMC8071103; doi:10.3390/pharmaceutics13040555)
Supplement: Supplementary file 1 [file pharmaceutics-13-00555-s001.pdf]

pharmaceutics

Supplementary Materials: Complying with the Guideline for Quality and Equivalence for Topical Semisolid Products: The Case of Clotrimazole Cream

Teresa Alves, Daniel Arranca, Ana Martins, Helena Ribeiro, Sara Raposo and Joana Marto

Table S1. Experimental design conditions, design and experimental matrices according to Central Composite Orthogonal (CCO) design.

ID	Design matrix		Experimental matrix	
	Cetyl palmitate	Octyldodecanol	Cetyl Palmitate (%, *w/w*)	Octyldodecanol (%, *w/w*)
F1	−1	−1	0.5	5
F2	1	−1	4	5
F3	−1	1	0.5	15
F4	1	1	4	15
F5	−1.14744 ($-\alpha$)	0	0.24198	10
F6	1.14744 (α)	0	4.25802	10
F7	0	−1.1744 ($-\alpha$)	2.25	4.2628
F8	0	1.14744 (α)	2.25	15.7372
F9	0	0	2.25	10
F10	0	0	2.25	10
F11	0	0	2.25	10

Table S2. Mathematical models used in the fitting of rheological data.

Model	Equation		Parameters
Power law	$\sigma = k\dot{\gamma}^{\eta}$	(S1)	σ–shear stress
Bingham	$\sigma = \sigma_0 + \eta_B\,\dot{\gamma}$	(S2)	σ_0–yield stress
Herschel-Bulkley	$\sigma = \sigma_0 + k\dot{\gamma}^{\eta}$	(S3)	γ–shear rate
Casson	$\sqrt{\sigma} = \sqrt{\sigma_0} + \sqrt{\eta_c\,\dot{\gamma}}$	(S4)	η_B–Bingham viscosity (or plastic viscosity)<br>k–consistency
Cross	$\frac{\eta - \eta_\infty}{\eta_0 - \eta_\infty} = \frac{1}{1 + (k\,\dot{\gamma})^m}$	(S5)	η_C–Casson viscosity<br>η and m–shear-thinning index[1]
Sisko	$\sigma = k\,\dot{\gamma}^{\eta} + \eta_\infty\dot{\gamma}$	(S6)	η_0–zero shear viscosity<br>η_∞–infinite shear viscosity

[1] if $\eta < 1$ material is shear-thinning, if $\eta > 1$ material is shear-thickening.

Table S3. Models used in DDSolver for fitting drug release data.

Model	Equation		Parameters
First-order	$F = 100 \times (1 - e^{-k_1 \times t})$	(S7)	k_1–first-order release constant
Higuchi	$F = k_H \times t^{0.5}$	(S8)	k_H–Higuchi release constant<br>k_{KP}–release constant
Korsmeyer-Peppas	$F = k_{KP} \times t^{n}$	(S9)	n–diffusional exponent indicating drug-release mechanism

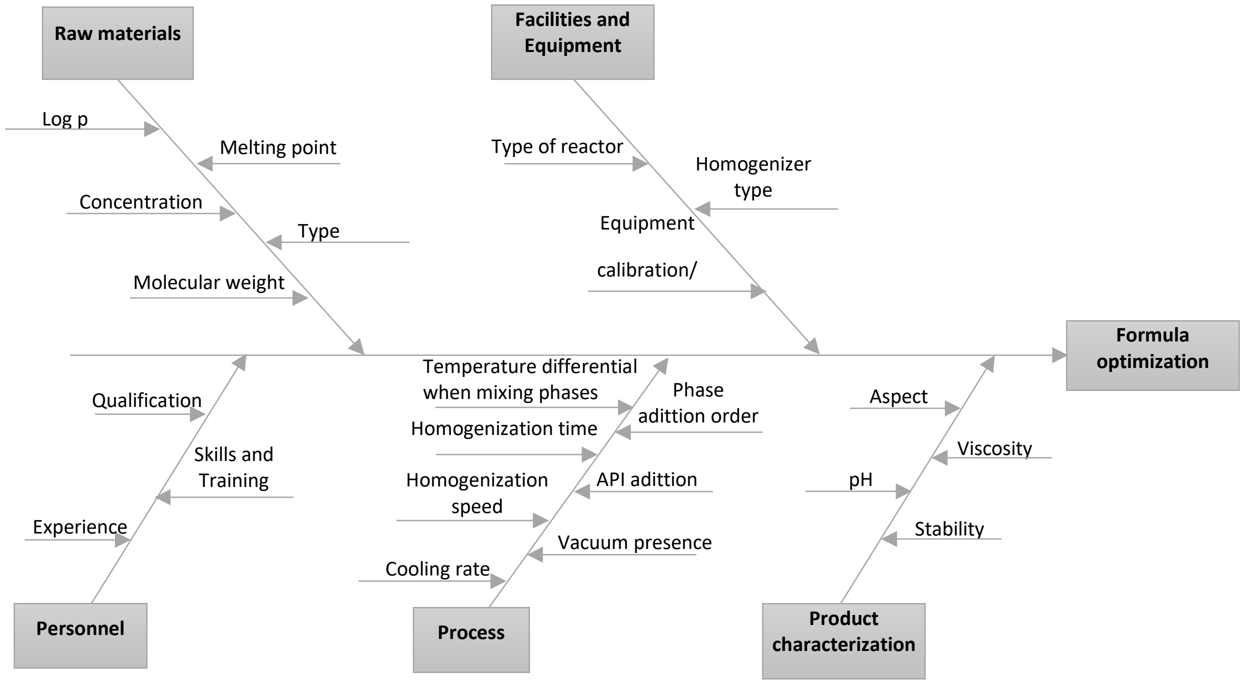

Figure S1. Ishikawa diagram to identify Critical Quality Attributes (CQAs).

Table S4. pH values of DoE (Design of Experiments) formulations and RF (reference formulation) at room temperature.

Samples	pH
F1	6.83
F2	7.04
F3	7.14
F4	7.31
F5	7.20
F6	7.60
F7	5.76
F8	6.69
F9	7.11
F10	6.99
F11	7.19
RF	5.73

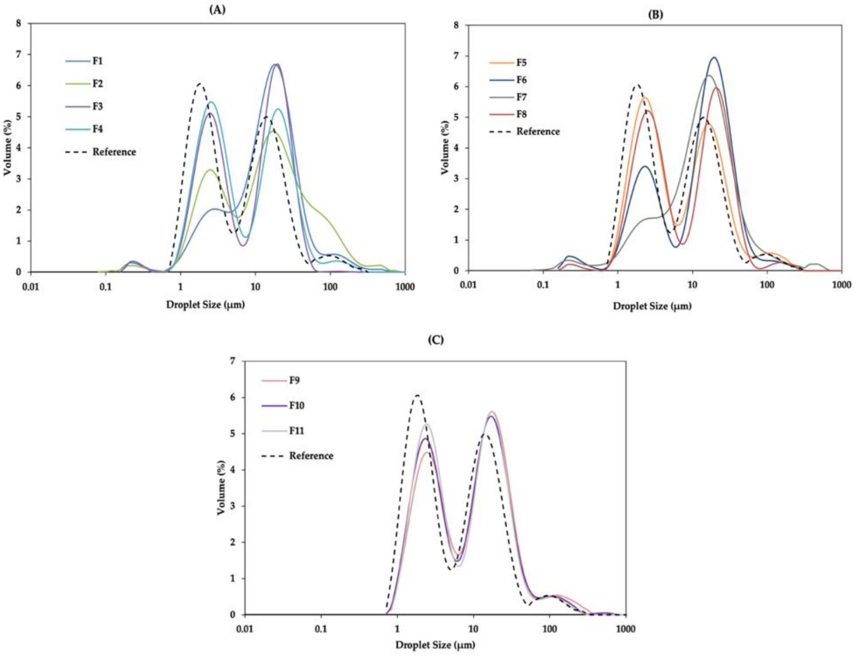

Figure S2. Droplet size distribution of DoE formulations and RF at room temperature: (**A**) F1–F4 and RF; (**B**) F5–F8 and RF; (**C**) F9–F11 and RF. The results shown are means, $n = 3$.

Table S5. Comparison of the effect of independent variables on cream rheological profile. Data are expressed as mean ± SD, $n = 3$.

Samples	Table of shear rate test	Frequency sweep test at 1 Hz		
	η_1 (Pa s)	G′ (Pa)	G″ (Pa)	Tan δ
F1	21.7 ± 0.7	2917.0 ± 38.2	1035.5 ± 19.1	0.35 ± 0.00
F2	46.6 ± 47.7	3673.0 ± 181.7	1073.0 ± 34.7	0.29 ± 0.01
F3	21.9 ± 1.6	1761.0 ± 107.5	537.2 ± 38.8	0.31 ± 0.00
F4	26.6 ± 2.5	1984.0 ± 205.8	589.7 ± 60.7	0.30 ± 0.00
F5	25.1 ± 7.9	1849.0 ± 189.5	577.4 ± 67.0	0.31 ± 0.01
F6	30.7 ± 6.4	2217.7 ± 144.9	701.6 ± 102.0	0.32 ± 0.00
F7	26.1 ± 0.5	2989.0 ± 127.3	949.3 ± 39.5	0.32 ± 0.00
F8	23.0 ± 0.5	2441.0 ± 701.4	775.8 ± 236.9	0.32 ± 0.01
F9	28.6 ± 6.7	2598.5 ± 78.5	787.6 ± 27.2	0.30 ± 0.00
F10	29.2 ± 6.4	2624.0 ± 245.4	788.0 ± 73.7	0.30 ± 0.00
F11	27.3 ± 4.2	2587.0 ± 272.9	788.4 ± 89.8	0.31 ± 0.00
RF	41.9 ± 14.9	2611.3 ± 171.3	522.9 ± 59.8	0.20 ± 0.01

G′—storage modulus, G″—loss modulus, η1—Viscosity at a shear rate 1 s^{-1}, Tan δ—loss factor (=G″/G′).

Table S6. Regression parameters from Bingham, Casson and Cross models fitted to the rheological data. Data are expressed as mean ± SD, *n* = 3.

Samples	Bingham			Casson			Cross				
	σ0	k	R^2	σ0	η_c	R^2	η_0	η_∞	k	η	R^2
F1	12.5 ± 2.1	0.6 ± 1.1	0.998 ± 0.00	6.6 ± 2.1	4.9 ± 1.2	0.996 ± 0.00	-	-	-	-	-
F2	27.4 ± 0.3	12.1 ± 0.2	0.976 ± 0.02	16.9 ± 0.7	5.5 ± 0.3	0.989 ± 0.01	-	-	-	-	-
F3	10.3 ± 2.4	10.6 ± 0.6	0.998 ± 0.00	5.2 ± 2.2	6.2 ± 1.3	0.999 ± 0.00	1633.0 ± 0.0	8.8 ± 0.0	207.5 ± 0.0	0.9 ± 0.0	0.999 ± 0.00
F4	17.7 ± 0.2	9.1 ± 3.3	0.972 ± 0.03	10.1 ± 1.1	4.5 ± 0.9	0.987 ± 0.00	4366.0 ± 0.0	7.3 ± 0.0	573.1 ± 0.0	0.8 ± 0.0	0.998 ± 0.00
F5	12.0 ± 4.6	9.4 ± 1.8	0.990 ± 0.01	6.7 ± 3.6	5.1 ± 1.8	0.993 ± 0.00	126.0 ± 0.0	12.9 ± 0.0	7.1 ± 0.0	1.5 ± 0.0	0.998 ± 0.00
F6	12.9 ± 3.0	10.4 ± 0.3	0.993 ± 0.01	6.8 ± 2.3	5.8 ± 0.7	0.994 ± 0.04	140.1 ± 0.0	12.2 ± 0.0	6.9 ± 0.0	1.3 ± 0.0	0.999 ± 0.00
F7	12.8 ± 2.3	10.9 ± 0.2	0.998 ± 0.00	6.4 ± 2.1	6.4 ± 0.8	0.997 ± 0.00	615.1 ± 619.3	10.4 ± 2.9	55.0 ± 66.7	1.0 ± 0.2	0.998 ± 0.00
F8	13.9 ± 1.3	11.6 ± 0.4	0.991 ± 0.00	6.9 ± 1.3	6.9 ± 0.3	0.994 ± 0.01	4643.9 ± 6282.2	10.1 ± 2.8	1476.5 ± 2078.1	0.9 ± 0.3	0.997 ± 0.00
F9	15.5 ± 2.9	10.6 ± 0.5	0.990 ± 0.01	8.2 ± 1.8	6.2 ± 0.3	0.993 ± 0.00	-	-	-	-	-
F10	15.0 ± 5.1	11.3 ± 1.6	0.993 ± 0.01	7.9 ± 3.9	6.5 ± 1.5	0.996 ± 0.01	213.6 ± 34.9	13.6 ± 1.5	10.9 ± 2.5	1.2 ± 0.1	0.999 ± 0.00
F11	12.9 ± 0.8	10.9 ± 0.8	0.998 ± 0.02	6.3 ± 1.0	6.7 ± 0.8	0.999 ± 0.01	591.4 ± 0.0	7.8 ± 0.0	50.1 ± 0.0	0.9 ± 0.0	0.996 ± 0.00
RF	30.1 ± 17.5	20.3 ± 5.7	0.988 ± 0.01	21.1 ± 16.8	7.6 ± 2.1	0.991 ± 0.01	990.0 ± 0.0	13.7 ± 0.0	58.3 ± 0.0	1.0 ± 0.0	0.999 ± 0.00

σ0—yield stress, k—consistency, R2—correlation coefficient, η_c—Casson viscosity, η_0—zero shear viscosity, η_∞—infinite shear viscosity, η—shear-thinning index.

Table S7. Regression parameters from Herschel-Bulkley, Power law and Sisko models fitted to the rheological data. Data are expressed as mean ± SD, *n* = 3.

Samples	Herschel-Bulkley				Power law			Sisko			
	σ0	k	η	R^2	k	η	R^2	η_∞	η	k	R^2
F1	10.5 ± 3.8	11.1 ± 3.2	0.9 ± 0.1	0.999 ± 0.00	22.1 ± 0.9	0.5 ± 0.1	0.995 ± 0.01	7.2 ± 0.7	0.9 ± 0.3	15.3 ± 0.9	0.999 ± 0.00
F2	13.8 ± 9.8	29.1 ± 11.4	0.6 ± 0.2	0.991 ± 0.00	44.7 ± 1.3	0.4 ± 0.1	0.948 ± 0.01	15.7 ± 0.2	2.0 ± 0.4	49.0 ± 24.2	0.995 ± 0.00
F3	7.8 ± 3.2	14.2 ± 1.5	0.8 ± 0.0	0.999 ± 0.00	22.5 ± 1.9	0.5 ± 0.1	0.992 ± 0.01	7.8 ± 0.4	0.7 ± 0.1	14.2 ± 1.3	0.999 ± 0.00
F4	8.9 ± 11.4	19.7 ± 9.9	0.7 ± 0.4	0.992 ± 0.00	28.2 ± 3.4	0.4 ± 0.2	0.969 ± 0.04	14.9 ± 4.9	0.6 ± 0.5	30.0 ± 18.8	0.998 ± 0.00
F5	9.2 ± 1.7	13.3 ± 2.2	0.8 ± 0.2	0.993 ± 0.01	23.2 ± 4.6	0.5 ± 0.2	0.971 ± 0.04	7.6 ± 2.2	0.8 ± 0.0	13.6 ± 4.6	0.998 ± 0.00
F6	10.3 ± 1.6	13.9 ± 1.8	0.8 ± 0.1	0.995 ± 0.01	25.3 ± 4.2	0.5 ± 0.1	0.978 ± 0.02	8.7 ± 0.0	0.9 ± 0.2	16.8 ± 5.6	0.991 ± 0.01
F7	10.8 ± 4.1	13.4 ± 2.7	0.9 ± 0.1	0.999 ± 0.00	25.2 ± 1.7	0.4 ± 0.1	0.967 ± 0.04	9.2 ± 1.2	0.8 ± 0.1	14.9 ± 0.2	0.999 ± 0.00
F8	9.9 ± 5.9	16.8 ± 5.7	0.8 ± 0.2	0.997 ± 0.00	26.9 ± 1.1	0.5 ± 0.0	0.962 ± 0.05	8.6 ± 2.9	0.6 ± 0.3	20.4 ± 9.9	0.999 ± 0.00
F9	9.9 ± 2.6	17.8 ± 6.5	0.8 ± 0.2	0.997 ± 0.00	28.5 ± 4.8	0.5 ± 0.1	0.971 ± 0.02	5.1 ± 3.2	0.6 ± 0.2	21.1 ± 0.5	0.999 ± 0.00
F10	10.8 ± 1.7	16.9 ± 2.9	0.8 ± 0.1	0.998 ± 0.00	28.5 ± 5.6	0.6 ± 0.1	0.999 ± 0.02	10.3 ± 2.6	1.1 ± 0.9	21.3 ± 10.6	0.997 ± 0.01
F11	5.2 ± 10.2	20.8 ± 12.3	0.7 ± 0.3	0.996 ± 0.00	26.3 ± 2.5	0.5 ± 0.1	0.952 ± 0.04	16.8 ± 15.0	0.7 ± 0.9	2.2 ± 18.7	0.995 ± 0.01
RF	20.9 ± 15.8	38.7 ± 22.1	0.6 ± 0.3	0.999 ± 0.00	54.1 ± 22.7	0.4 ± 0.1	0.994 ± 0.00	12.9 ± 6.1	0.5 ± 0.1	59.9 ± 35.9	0.999 ± 0.00

Table S8. Obtained values from *in* vitro release studies of DoE formulations and RF after 12 h. Data are expressed as mean ± SD, *n* = 3.

Samples	Release $_{12\,h}$ (%)	DE $_{12\,h}$ (%)	AUC
F1	9.6 ± 0.2	4.8 ± 0.1	85.8 ± 5.1
F2	9.2 ± 0.3	4.6 ± 0.1	78.5 ± 2.4
F3	25.8 ± 0.3	12.9 ± 0.1	204.4 ± 12.1
F4	22.5 ± 0.1	11.3 ± 0.0	171.2 ± 6.6
F5	22.9 ± 0.3	11.5 ± 0.1	184.1 ± 6.7
F6	24.7 ± 0.2	12.3 ± 0.1	192.9 ± 1.8
F7	10.3 ± 0.4	5.1 ± 0.2	86.7 ± 0.8
F8	28.5 ± 0.3	14.2 ± 0.1	221.3 ± 11.6
F9	23.9 ± 0.3	11.9 ± 0.1	193.1 ± 8.3
F10	24.0 ± 0.8	12.0 ± 0.4	184.2 ± 5.1
F11	24.2 ± 0.6	12.1 ± 0.3	189.4 ± 6.6
RF	23.9 ± 0.4	11.9 ± 0.2	189.4 ± 2.6

DE—dissolution efficiency, AUC—area under the curve.

Table S9. Regression coefficients obtained by fitting First-order, Higuchi and Korsmeyer-Peppas mathematical models to the release data from DoE formulations and RF after 12 h. Data are expressed as mean ± SD, $n = 3$.

Samples	First-order			Higuchi			Korsmeyer-Peppas			
	k1	R^2	AIC	k_H	R^2	AIC	k_{KP}	n	R^2	AIC
F1	0.01 ± 0.0	0.311 ± 0.33	27.4 ± 3.8	3.1 ± 0.2	0.880 ± 0.10	10.9 ± 12.9	4.6 ± 1.1	0.3 ± 0.1	0.999 ± 0.00	19.8 ± 7.1
F2	0.01 ± 0.0	0.592 ± 0.12	23.9 ± 1.8	2.8 ± 0.1	0.972 ± 0.02	3.0 ± 7.1	3.5 ± 0.4	0.4 ± 0.1	0.998 ± 0.00	12.9 ± 4.1
F3	0.03 ± 0.0	0.883 ± 0.08	29.7 ± 4.8	7.5 ± 0.4	0.977 ± 0.02	17.2 ± 7.5	6.5 ± 1.5	0.6 ± 0.1	0.988 ± 0.01	12.4 ± 10.3
F4	0.03 ± 0.0	0.912 ± 0.03	26.3 ± 2.4	6.3 ± 0.2	0.981 ± 0.01	14.9 ± 4.6	5.1 ± 0.6	0.6 ± 0.1	0.994 ± 0.00	5.9 ± 7.8
F5	0.03 ± 0.0	0.825 ± 0.04	31.3 ± 1.3	6.7 ± 0.2	0.994 ± 0.01	6.6 ± 6.6	6.6 ± 0.5	0.5 ± 0.0	0.994 ± 0.01	5.4 ± 9.3
F6	0.03 ± 0.0	0.884 ± 0.01	29.7 ± 0.7	7.1 ± 0.1	0.992 ± 0.00	10.5 ± 4.6	6.3 ± 0.3	0.6 ± 0.0	0.996 ± 0.00	4.1 ± 5.9
F7	0.01 ± 0.0	0.672 ± 0.11	24.2 ± 1.8	3.1 ± 0.0	0.984 ± 0.01	2.4 ± 3.6	3.6 ± 0.4	0.4 ± 0.1	0.996 ± 0.00	11.1 ± 11.1
F8	0.03 ± 0.0	0.904 ± 0.05	29.9 ± 4.1	8.1 ± 0.4	0.979 ± 0.01	18.2 ± 7.5	6.9 ± 1.2	0.6 ± 0.1	0.989 ± 0.01	11.3 ± 10.9
F9	0.03 ± 0.0	0.744 ± 0.12	33.4 ± 3.7	7.0 ± 0.2	0.981 ± 0.01	15.4 ± 2.7	7.7 ± 1.5	0.5 ± 0.1	0.992 ± 0.00	9.2 ± 5.5
F10	0.03 ± 0.0	0.781 ± 0.04	32.5 ± 0.9	6.7 ± 0.2	0.979 ± 0.01	15.8 ± 2.6	7.1 ± 0.5	0.5 ± 0.0	0.978 ± 0.01	16.8 ± 3.4
F11	0.03 ± 0.0	0.789 ± 0.07	32.5 ± 2.0	6.9 ± 0.2	0.979 ± 0.01	16.3 ± 2.5	7.2 ± 0.9	0.5 ± 0.1	0.979 ± 0.01	16.3 ± 4.3
RF	0.03 ± 0.0	0.530 ± 0.08	28.5 ± 0.3	6.9 ± 0.1	0.981 ± 0.01	7.7 ± 6.1	7.3 ± 0.2	0.5 ± 0.0	0.982 ± 0.02	6.8 ± 8.4

K is the release rate constant; *b* is the intercept and R^2 the coefficient of determination.

Table S10. Summary of ANOVA parameters concerning the fitted model's characterization.

CQAs	Regression			Lack of fit	
	F_1	Prob > F	R^2	F_2	Prob > F
Viscosity	10.98	0.005	0.825	13.60	0.070
G′	9.38	0.008	0.801	367.53	0.003
G′′	7.55	0.013	0.764	-	-
Release $_{12\,h}$	58.90	0.000	0.936	171.82	0.006
DE $_{12\,h}$	58.98	0.000	0.936	170.73	0.006
Permeation $_{24\,h}$	19.38	0.002	0.683	75.91	0.013
Retention	7.24	0.015	0.756	3.20	0.255

Table S11. Summary of regression analysis results for measured responses.

CQAs	Regression	SS	EmoA	SS × SS	EmoA × EmoA	SS × EmoA
Viscosity	Coeff value	0.08 ± 0.02	−0.05 ± 0.02	-	-	−0.06 ± 0.02
	Prob	0.003	0.038	-	-	0.029
G′	Coeff value	0.04 ± 0.02	−0.09 ± 0.02	−0.06 ± 0.03	-	-
	Prob	0.108	0.003	0.063	-	-
G′′	Coeff value	0.02 ± 0.02	−0.09 ± 0.02	−0.06 ± 0.0.3	-	-
	Prob	0.342	0.004	0.119	-	-
Release $_{12\,h}$	Coeff value	-	7.61 ± 0.78	-	−5.16 ± 1.08	-
	Prob	-	0.000	-	0.001	-
DE $_{12\,h}$	Coeff value	-	3.80 ± 0.39	-	−2.58 ± 0.54	-
	Prob	-	0.000	-	0.001	-
Permeation $_{24\,h}$	Coeff value	-	0.79 ± 0.18	-	-	-
	Prob	-	0.002	-	-	-
Retention	Coeff value	−0.13 ± 0.10	0.41 ± 0.10	0.27 ± 0.14	-	-
	Prob	0.240	0.005	0.093	-	-

SS–secondary surfactant; EmoA–emollient A.

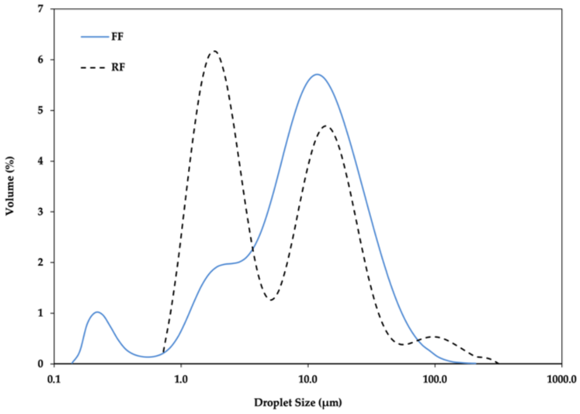

Figure S3. Droplet size distribution of FF and RF stored at room temperature. The results shown are means, n = 3.

Table S12. Regression parameters from Bingham, Casson, Cross, Herschel-Bulkley, Power law and Siso models fitting to the rheological data. Data are expressed as mean ± SD, n = 3.

Models		Samples	
		FF	RF
Bingham	σ_0	20.4 ± 7.9	30.1 ± 17.5
	k	12.9 ± 1.1	20.3 ± 5.7
	R^2	0.995 ± 0.00	0.988 ± 0.01
Casson	σ_0	11.7 ± 6.8	21.1 ± 16.8
	η_c	6.9 ± 1.6	7.6 ± 2.1
	R^2	0.997 ± 0.00	0.991 ± 0.01
Cross	η_0	11,590.0 ± 0.0	990.0 ± 0.0
	η_∞	8.4 ± 0.0	13.7 ± 0.0
	k	1783.0 ± 0.0	58.3 ± 0.0
	η	0.8 ± 0.0	1.0 ± 0.0
	R^2	0.998 ± 0.0	0.999 ± 0.0
Herschel-Bulkley	σ_0	16.9 ± 6.4	20.9 ± 15.8
	k	17.7 ± 3.6	38.7 ± 22.1
	η	0.8 ± 0.1	0.6 ± 0.3
	R^2	0.999 ± 0.00	0.999 ± 0.00
Power law	k	35.9 ± 9.5	54.1 ± 22.7
	η	0.4 ± 0.2	0.4 ± 0.1
	R^2	0.973 ± 0.03	0.994 ± 0.00
Sisko	η_∞	10.9 ± 0.9	12.9 ± 6.1
	η	0.8 ± 0.1	0.5 ± 0.1
	k	23.7 ± 10.3	59.9 ± 35.9
	R^2	0.999 ± 0.01	0.999 ± 0.01

σ_0 is yield stress, k is consistency, η is shear-thinning index, η_0 is zero shear viscosity, η_∞ is infinite shear viscosity and η_c is Casson viscosity. FF is final formulation, RF is reference formulation.

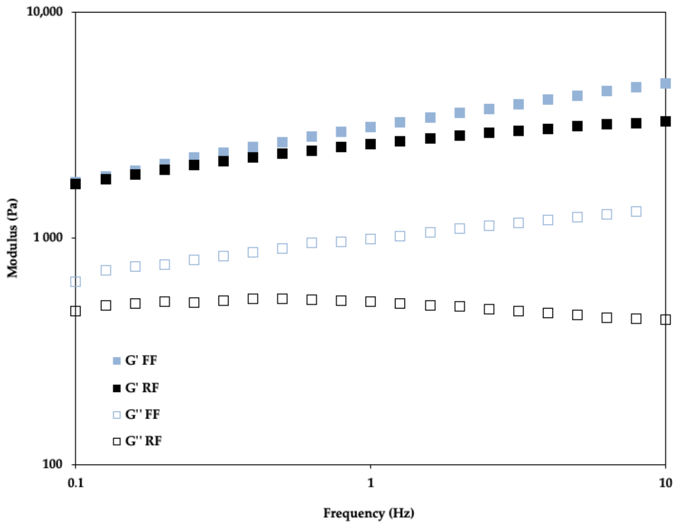

Figure S4. Storage (G′) and loss (G′′) moduli of the FF and RF. Data shown are means, *n* = 3.

Table S13. Regression coefficients obtained from the fitting of First-order, Higuchi and Korsmeyer-Peppas mathematical models to the release data from the FF and RF. Data are mean ± SD, *n* = 6.

Models		Samples	
		**FF**	**RF**
First-order	k1	0.02 ± 0.01	0.02 ± 0.01
	R^2	0.39 ± 0.58	0.59 ± 0.10
	AIC	25.4 ± 3.3	24.5 ± 6.1
Higuchi	k_H	5.68 ± 1.03	5.8 ± 1.6
	R^2	0.89 ± 0.18	0.98 ± 0.01
	AIC	12.5 ± 8.9	3.6 ± 7.2
Korsmeyer-Peppas	k_{KP}	5.87 ± 0.62	5.88 ± 2.02
	n	0.48 ± 0.11	0.49 ± 0.04
	R^2	0.91 ± 0.11	0.99 ± 0.00
	AIC	10.6 ± 12.2	1.7 ± 9.4

k1, k_H and k_{KP} are the release rate constants and R^2 the coefficient of determination.
